# New tools to convert bacterial artificial chromosomes to a self-excising design and their application to a herpes simplex virus type 1 infectious clone

**DOI:** 10.1186/s12896-016-0295-4

**Published:** 2016-08-31

**Authors:** Alexsia L. Richards, Patricia J. Sollars, Gregory A. Smith

**Affiliations:** 1 Department of Microbiology-Immunology, Northwestern University Feinberg School of Medicine, 303 E. Chicago Ave., Morton Building, Room 3-603, Chicago, IL 60611 USA; 2 School of Veterinary Medicine and Biomedical Sciences, University of Nebraska, Lincoln, NE 68583 USA

**Keywords:** Bacterial artificial chromosome, BAC, Self-excising, Self-recombining, Herpesvirus, Herpes simplex virus type 1, HSV-1, Infectious clone

## Abstract

**Background:**

Infectious clones are fundamental tools for the study of many viruses, allowing for efficient mutagenesis and reproducible production of genetically-defined strains. For the large dsDNA genomes of the herpesviridae, bacterial artificial chromosomes have become the cloning vector of choice due to their capacity to house full-length herpesvirus genomes as single contiguous inserts. Furthermore, while maintained as plasmids in *Escherichia coli*, the clones can be mutated using robust prokaryotic recombination systems. An important consideration in the design of these clones is the means by which the vector backbone is removed from the virus genome upon delivery into mammalian cells. A common approach to vector excision is to encode loxP sites flanking the vector sequences and rely on Cre recombinase expression from a transformed cell line. Here we examine the efficiency of vector removal using this method, and describe a “self-excising” infectious clone of HSV-1 strain F that offers enhancements in virus production and utility.

**Results:**

Insertion of a fluorescent protein expression cassette into the vector backbone of the HSV-1 strain F clone, pYEbac102, demonstrated that 2 serial passages on cells expressing Cre recombinase was required to achieve > 95 % vector removal from the virus population, with 3 serial passages resulting in undetectable vector retention. This requirement was eliminated by replacing the reporter coding sequence with the CREin gene, which consists of a Cre coding sequence disrupted by a synthetic intron. This self-excising variant of the infectious clone produced virus that propagated with wild-type kinetics in culture and lacked vector attenuation in a mouse neurovirulence model.

**Conclusion:**

Conversion of a herpesvirus infectious clone into a self-excising variant enables rapid production of viruses lacking bacterial vector sequences, and removes the requirement to initially propagate viruses in cells that express Cre recombinase. The self-excising bacterial artificial chromosome described here allows for efficient production of the F strain of herpes simplex virus type 1.

**Electronic supplementary material:**

The online version of this article (doi:10.1186/s12896-016-0295-4) contains supplementary material, which is available to authorized users.

## Background

Herpes simplex virus type 1 (HSV-1) infections are typically benign, with cold sores (herpes labialis) being the most common form of disease [1]. Rarer, but more severe disease outcomes have a significant health toll due to the high prevalence of HSV-1 in the general population. For example, HSV-1 is the leading cause of infectious blindness and sporadic encephalitis in the United States [2, 3]. Understanding the molecular mechanisms underlying viral pathogenesis is essential for the development of new therapies to treat and possibly prevent infection. To this end, the cloning of the HSV-1 genome into a mini F plasmid as a bacterial artificial chromosome (BAC) allows for efficient production and mutagenesis of the virus using prokaryotic recombination systems [4–9]. While the BAC vector allows for stable maintenance of the clone in *E. coli*, its presence in the viral genome following transfection into mammalian cells can lead to instability and viral propagation defects [10]. To remove the BAC vector from the viral genome, a common solution is to flank the BAC sequences with loxP sites in direct orientation [7–9]. These designs require transfection of the BAC into mammalian cells expressing Cre recombinase, which imposes constraints when working with these clones. For example, transfection of BACs encoding lethal mutations requires simultaneous expression of Cre recombinase and the viral gene for trans-complementation. The aim of the present study was to streamline the conversion of floxed BAC clones to self-excising BACs, and thereby eliminating the need for Cre-expressing cells and concomitant monitoring of vector removal [8, 11–13]. Using a HSV-1 BAC, we demonstrate that modification to a self-excision design avoids concerns of inadvertently working with partially excised virus populations, which we demonstrate can result in attenuation of neurovirulence.

## Methods

### Production of the pEP-TagBFP-in and pEP-CREin-in En Passant plasmids

The TagBFP expression cassette from pTagBFP-C (Evrogen) was subcloned into pEGFP-S1 to move the cassette into an ampicillin resistant vector backbone. This resulted in pGS4742. A fragment of the TagBFP ORF was amplified from pGS4742 using primers GS835: 5′-GTTTGACTCACGGGGATTTCC (anneals within the CMV IE promoter) and GS4715: 5′- GGCAGCTGGGATCCGAGGTTCTTAGCGGGTTTCTTGG (anneals within the TagBFP coding sequence; PvuII and BamHI palindromes are underlined) The PCR product was cloned into pGS4742 using a NheI site downstream the GS835 sequence, and a StuI site within the TagBFP ORF to receive the PvuII site encoded in GS4715, resulting in pGS4751. pGS4751 encodes a partial duplication of the TagBFP ORF with the duplicated sequences flanking the BamHI site derived from GS4715. The I-SceI site + kanamycin cassette from pEP-EGFP-in [14] was inserted into the BamHI site, resulting in the En Passant template plasmid, pEP-TagBFP-in (pGS4794).

The CREin cassette from pGS403 [11] was amplified using primers GS1495: 5′GGCCGCGGTAATACGACTCACTATAGGGC (SacII palindrome is underlined) and GS1496: 5′-GGAGATCTGAATTCCATGAGTGAACGAACC (BglII and EcoRI palindromes are underlined). The PCR product was cloned into pGS403, resulting in pGS1511. pGS1511 encodes a partial duplication of the CREin sequence, with the duplicated sequences flanking the EcoRI site derived from GS1496. Primers GS1513: ‘5-GGGAATTCTAGCTAGGGATAACAGG and GS1498: CCGAATTCTAGCCAGTGTTACAACC (EcoRI palindromes underlined) were used to amplify the I-SceI site + kanamycin cassette from pEP-EGFP-in [14]. The product was inserted into the EcoRI site resulting in the En Passant template plasmid, pEP-CREin-in (pGS1518).

### Virus construction

**Fig. 1:**
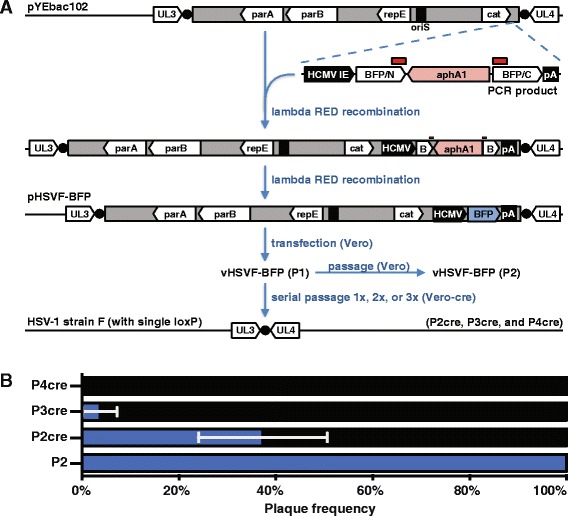
Construction and analysis of pHSVF-BFP. **a** Flow diagram of two-step recombination (En Passant) used to insert a TagBFP expression cassette into the vector backbone of pYEbac102 in *E. coli*, and subsequent Cre-based removal of the BAC vector from the viral genome in mammalian cells. The expression cassette was PCR amplified from the pEP-TagBFP-in template and recombined into the BAC vector backbone by lambda RED recombination using kanamycin resistance as selective pressure. In a second recombination step, the kanamycin resistance gene (aphA1) was removed based on partially duplicated sequences in the flanking TagBFP coding sequence (*red boxes*), which simultaneously established the contiguous BFP coding sequence and resulted in pHSVF-BFP. Transfection of pHSVF-BFP into Vero cells produced the vHSVF-BFP virus that stably expressed blue fluorescence, which was further expanded by a second passage (P2) on Vero cells. Transfection and serial passage in Vero-cre cells produced HSV-1 lacking the BAC backbone and associated fluorescence but retaining a single loxP site (*black circles*) between the UL3 and UL4 genes. **b** Excision of the pBeloBAC vector from vHSVF-BFP was monitored by fluorescent plaque assay. Following transfection of Vero cells (P1), vHSVF-BFP was successively passaged on Vero-cre cells for a second (P2cre), third (P3cre), and fourth round (P4cre), or on Vero cells that did not express Cre recombinase for a second round (P2). Plaques produced from each harvest were scored as positive (*blue*) or negative (*black*) for fluorescence. The data are a composite of three independent experiments consisting of >40 plaques scored per experiment. *Error bars* are SD

All HSV-1 recombinant viruses were derived from the pYEbac102 infectious clone of HSV-1 strain F [9]. pHSVF-BFP was generated by En Passant mutagenesis of the pYEbac102 infectious clone [15]. Recombination was performed in the Escherichia coli strain GS1783, which encodes inducible Red and I-SceI activities [15], following PCR amplification of the recombination fragment from the pEP-TagBFP-in template (primers used are listed in the Additional file 1: Table S1). The resulting HSV-1 infectious clone contained the TagBFP expression cassette in the BAC vector backbone (see Fig. 1). Likewise, pHSVF-CREin was produced through En Passant mutagenesis of the pHSVF-BFP infectious clone using the pEP-CREin-in template (primers used are listed in the Additional file 1: Table S1). Viruses were produced by electroporation of infectious clones into either Vero or HEK293T cells as previously described [16]. Cells were maintained in Dulbecco modified Eagle medium (DMEM) (Invitrogen) supplemented with 10 % bovine growth supplement (BGS) (Vero cells) or 10 % fetal bovine serum (FBS) (HEK293T cells). Serum levels were reduced to 2 % BGS/FBS approximately 12 h after electroporation. Virus was harvested at a time at which 100 % of the cells displayed pronounced cytopathic effect (CPE) (typically 3–5 days post electroporation). The harvested virus was passaged on either Vero or Vero-Cre cells as indicated.

### Viral propagation kinetics

Vero cells were seeded in 6-well trays and infected the next day at a multiplicity of infection (MOI) of 10. Following absorption of the virus for 1 h at 37 °C, the inoculum was removed and replaced with 2 ml of citrate (pH 3.0) for 1 min at RT to inactivate extracellular virions, and then washed 3 times with DMEM supplemented with 10 % FBS. Cells were then incubated in 2 ml of DMEM supplemented with 10 % FBS at 37 °C until the desired harvest time point. Both cell-associated and extracellular virus was collected as previously described [17]. Titers of all time points were determined by plaque assay on Vero cells and plotted with GraphPad Prism 4.

### Isolation of nucleocapsid DNA

Five 15 cm dishes of confluent Vero cells were infected at a MOI of 7. At 24 h post infection the media was removed and replaced with 2 ml PBS/dish. Cells were collected and combined into one 50 ml conical tube and pelleted at 750 × g for 10 min at RT. Cells were then washed once in PBS and resuspended in 10 ml LCM buffer 1 (1M KCl, 1M Tris [pH 7.4], 1M EDTA, 0.5 % NP-40). Cells were extracted twice with 1.5 ml Freon (1,1,2-trichloro-1,1,2-trifluoroethane) and the top layer collected and placed in a new conical. The following two-step gradient was prepared in a Seton 7030 centrifuge tube: bottom layer, 2.5 ml LCM buffer 2 (1M KCl, 1M Tris [pH 7.4], 1M EDTA, 0.5 % NP-40, 45 % glycerol), top layer, 3.0 ml LCM buffer 3 (1M KCl, 1M Tris [pH 7.4], 1M EDTA, 0.5 % NP-40, 5 % glycerol). The cell extract was split in half and layered on top of two gradients. Gradients were centrifuged at 25,000 rpm for 1 h in a SW41 rotor at 4 °C. Following centrifugation, fluid was aspirated from the tube and the pellet was resuspended in 0.5 ml TNE (0.5M EDTA, 5M NaCl, 1M Tris-Base [pH 7.5]) and transferred to a 50 ml conical. 8.5 ml of TNE, 0.5 ml of 10 % SDS and 0.2 mg of proteinase K was added and the conical inverted several times to mix. DNA was extracted twice with phenol/chloroform, followed by overnight precipitation in 30 ml of −20 °C ethanol and 4.5 ml of 3M sodium acetate at −80 °C. Following precipitation DNA was isolated by centrifugation at 14,000 × g for 30 min at 4 °C. The DNA pellet was washed once with 4 °C 70 % ethanol, dried, and resuspended in 0.5 ml TNE. For PCR analysis of BAC vector excision, two primer pairs were used. Primer pair A was GS5840 and GS5841 (Additional file 1: Table S1), and primer pair B was: GS6417 (5′ GCATTTCCTCGTGGCGAAT) and GS6418 (5′ GTCCCCTTACAGTTCCACC).

### Analysis of plaque florescence

Expression of BFP was assessed by plaque assay, following 10-fold dilutions of virus stocks on Vero cells in 6-well trays. Following virus absorption, cells were overlaid with DMEM containing 2 % methocel and 2 % BGS. At 4 days post infection images were captured with a 4× objective on a TE2000 inverted fluorescence microscopy (Nikon) fitted with a CoolSnap HQ2 camera (Photometrics). A total of three independent experiments were conducted with a minimum of 40 plaques scored for BFP emissions per virus per experiment. The data from all three experiments were combined and plotted in Prism 4 (GraphPad).

### In vivo inoculation of virus

All procedures conformed to NIH guidelines for work with laboratory animals and were approved by the Institutional Animal Care and Use Committee of the University of Nebraska, Lincoln. Male CD-1 mice (6 weeks old; Charles River) were maintained for at least 2 weeks under a 12:12 h light/dark cycle, two to three mice per cage with food and water freely available. Intracranial application of virus was administered to animals anesthetized by 2.5–5.0 % isoflurane inhalation. Viral stocks were maintained frozen at −80 °C and used immediately after being thawed. Each animal received 1.9 × 10^5^ PFU directly injected into the brain. All animals received injections 1 mm lateral, 1 mm caudal of bregma, while the injections were placed 2 mm ventral of the dura. Behavior was continuously video monitored with images captured every 10 min. Survival times post-inoculation were rounded to the nearest hour.

## Results

### Efficiency of Cre-mediated BAC vector excision

The pYEbac102 infectious clone of HSV-1 strain F is based upon a pBeloBAC vector backbone flanked by a pair of loxP sites, which is inserted between the UL3 and UL4 genes of the virus genome [9]. As a first step to producing a self-excising variant of pYEbac102, a suitable site for insertion of a mammalian expression cassette was identified that did not disrupt BAC replication, partitioning, or antibiotic-resistance functions in *E. coli*, but also achieved robust expression in mammalian cells. To monitor mammalian expression, a cassette consisting of the coding sequence of a blue fluorescent protein (TagBFP) driven by the HCMV immediate early promoter (HCMV IE), and flanked by a SV40 polyadenylation sequence (pA), was initially inserted downstream of the chloramphenicol acetyl transferase gene (cat) that provides antibiotic selectivity in the BAC vector backbone of pYEbac102. For this purpose, an En Passant template plasmid, pEP-TagBFP-in, was produced and primers were designed to amplify the cassette with 5′ ends homologous to the BAC sequences flanking the site of insertion (Fig. 1a) [14, 15]. The new BAC was successfully maintained in *E. coli* under chloramphenicol selection, and was designated pHSVF-BFP.

Transfection of pHSVF-BFP into Vero cells resulted in productive infection with corresponding expression of fluorescence. Nascent virus was isolated, termed vHSVF-BFP, and either expanded once on Vero cells or serial passaged in Vero cells stably expressing Cre recombinase [7]. vHSVF-BFP that was not exposed to Cre recombinase consistently produced fluorescent plaques (Fig. 1b). A single expansion of vHSVF-BFP on Vero-cre cells resulted in approximately 60 % of the resulting PFU lacking BFP expression during subsequent infection of Vero cells, with two additional serial passages on Vero-cre cells needed to eliminate BFP-expressing virus. The loss of fluorescence was consistent with loxP recombination and removal of the BAC vector sequence from the HSV-1 genome (see below). These results indicate that multiple serial passages were required to produce a genetically homogenous virus population that lacked the BAC vector backbone as a result of loxP recombination mediated by Cre recombinase.

### Production of a self-excising infectious clone variant

**Fig. 2:**
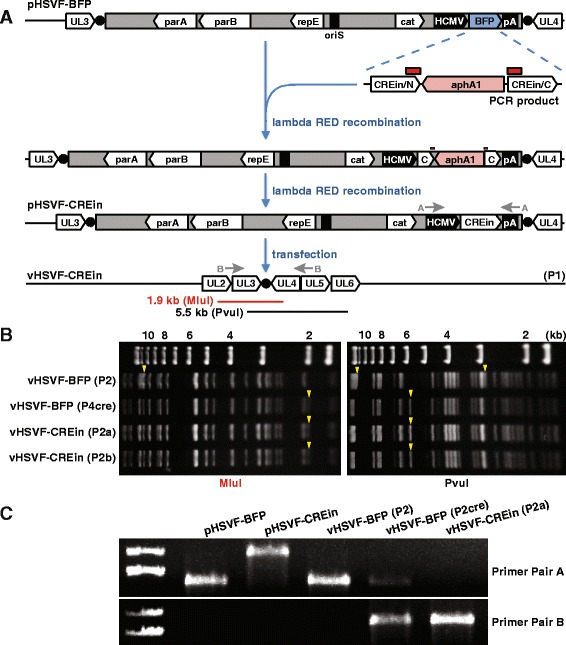
Construction and analysis of pHSVF-CREin. **a** The infectious clone pHSVF-CREin was made by insertion of the CREin expression cassette into the pHSVF-BFP infectious clone in a process paralleling that described in Fig. 1. The CREin expression cassette was PCR amplified from the pEP-CREin-in template and recombined into pHSVF-BFP BAC vector backbone by lambda RED recombination, resulting in the replacement of the TagBFP coding sequence with that of CREin::kanamycin. In the second recombination step, the kanamycin resistance gene (aphA1) was removed based on partially duplicated sequences in the flanking CREin coding sequence (*red boxes*), which simultaneously established the contiguous CREin coding sequence and resulted in pHSVF-CREin. Excision of the BAC from pHSVF-CREin was achieved by autonomous expression of Cre recombinase following introduction of the DNA into either Vero or HEK293T cells, resulting in a passage 1 (P1) harvest. **b** MluI (*left*) or PvuI (*right*) restriction analysis of HSV-1 DNA harvested from purified nucleocapsids. *Yellow arrow heads* indicate restriction fragments that mark the presence or absence of the BAC vector sequences. Size standards are indicated in kb. **c** Amplification of viral and plasmid DNA using primers designed to detect removal of BAC DNA from the viral genome. The positions of the primer pairs are indicated in panel A

Self-excising BAC clones function through expression of Cre recombinase from the vector backbone during infection of mammalian cells [8, 11–13]. Because insertion of the TagBFP expression cassette downstream of the cat gene (Fig. 1a) did not detectably interfere with BAC maintenance in *E. coli* and provided effective expression during infection of mammalian cells (Fig. 1b), we modified the cassette to express Cre recombinase. Specifically, the TagBFP coding sequence was replaced with the CREin gene while maintaining the HCMV promoter and SV40 polyadenylation sequences (Fig. 2a). CREin is a Cre recombinase coding sequence that is disrupted by a synthetic intron, thereby preventing leaky expression in *E. coli* [11]. For this purpose, an En Passant template plasmid was produced, pEP-CREin-in, and primers were designed to amplify the gene with 5′ ends homologous to the upstream HCMV promoter and downstream SV40 polyadenylation sequences flanking the site of insertion. Recombination was carried out by the En Passant procedure as described above. The new BAC, pHSVF-CREin, was transformed, harvested, and expanded one additional time on Vero cells to produce high-titer stocks. This procedure was performed twice to produce two independent stocks of the vHSVF-CREin virus: passage 2a (P2a) passage 2b (P2b).

Restriction enzyme digestion of DNA isolated from HSV-1 nucleocapsids was used to examine the virus genome for the presence of the BAC vector backbone. DNA from vHSVF-BFP (P2) served as a positive control and vHSVF-BFP (P4cre) provided the negative control (Fig. 1b). Digestion of nucleocapsid DNA with MluI indicated that the 10.5 kb BAC-derived fragment observed in vHSVF-BFP (P2) was replaced by a 1.9 kb band in vHSVF-BFP (P4cre), vHSVF-CREin (P2a), and vHSVF-CREin (P2b), demonstrating self-excision following transfection of pHSVF-CREin into mammalian cells (Fig. 2b). This result also provided confirmation that BAC excision accounted for the loss of fluorescence in vHSVF-BFP (P4cre) (Fig. 1b). Similarly, digestion of vHSVF-BFP (P2) DNA with PvuI resulted in 11.3 and 2.7 kb BAC derived fragments, which were replaced by a 5.5 kb band in vHSVF-BFP (P4cre), vHSVF-Cre (P2a), and vHSVF-Cre (P2b) (Fig. 2b).

The nucleocapsid DNA was inspected further by PCR assay using primers designed to anneal to sequences within the BAC vector (Fig. 2c). DNA from the pHSVF-BFP and pHSVF-CREin plasmids served as controls. The size difference observed in the PCR products between pHSVF-BFP and pHSVF-CREin reflects the larger size of the CREin gene relative to the BFP coding sequence. As expected, primer pair A, which detects the region of the BAC vector backbone containing the introduced expression cassette, amplified an equivalent fragment from pHSVF-BFP and vHSVF-BFP (P2). Whereas residual BAC vector was detected from HSV-1 genomic DNA isolated from vHSVF-BFP (P2cre), this was not the case with HSV-1 produced from the pHSVF-CREin self-excising clone: vHSVF-CREin (P2a). Primer pair B produced results supporting this interpretation, indicating that excision occurred when providing Cre recombinase in trans or from the BAC backbone (primer pair B did not yield a product from the two BAC plasmids or vHSVF-BFP presumably due to the 18 kb distance between the primer annealing sites accounted for by the presence of the BAC vector backbone). Finally, the analysis of vHSVF-BFP nucleocapsid DNA described here confirms that the loss of fluorescence observed in Fig. 1b was due to Cre-loxP recombination.

### Propagation kinetics and neurovirulence of HSV-1 derived from the self-excising BAC

**Fig. 3:**
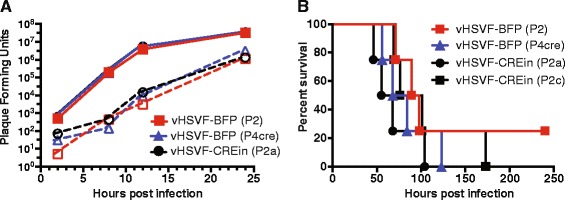
Comparison of virus propagation and neurovirulence. **a** Single-step growth curves comparing propagation of unexcised vHSVF-BFP (P2), excised vHSVF-BFP (P4cre), and self-excised vHSVF-CREin (P2a). Virus was harvested from cell media (*dashed lines*) and cells (*solid lines*) and titers were measured by plaque assay. **b** Kaplan–Meier presentation of mouse survival following intracranial inoculation of unexcised vHSVF-BFP (P2), excised vHSVF-BFP (P4cre), and two isolates of self-excised vHSVF-CREin (P2a and P2c)

The replication kinetics of the vHSVF-CREin stock (P2a) was indistinguishable from vHSVF-BFP (P2) and vHSVF-BFP (P4cre) (Fig. 3a). These results indicate that the self-excision process did not result in unexpected attenuation of virus growth. When viruses were administered intracerebrally into CD-1 mice, the presence of the BAC vector in the HSV-1 genome slightly reduced virulence, with some animals surviving beyond 200 hpi (Fig. 3b). As expected, isolates of HSV-1 derived from the self-excising BAC clone killed all mice before the 200 hpi time point, consistent with a manually excised virus that had been serial passaged for three rounds in Vero-Cre cells and verified by fluorescent plaque assay to be fully excised.

## Discussion

Herpesvirus infectious clones are important tools for researchers studying these large viruses. Following mutagenesis in *E. coli*, transfection of BAC clones into mammalian cells results in productive infections that yield virus populations that uniformly harbor the desired genetic alterations. While the utility of BAC infectious clones has made them the preferred means to genetically manipulate herpesviruses since their initial development [18], one ongoing issue is the need to efficiently remove the BAC vector from the viral genome upon delivery into mammalian cells. A common practice is to design BAC clones with loxP sites flanking the BAC vector backbone and supply Cre recombinase in trans in mammalian cells. Thus, the BAC is excised from the virus genome with a single loxP site remaining as the only foreign sequence. Because the viruses studied are only as good as the infectious clone from which they are derived, placing the “floxed” BAC vector, and thereby ultimately the residual loxP site, in an innocuous site in the genome is a critical design consideration. By contrast, the means by which the BAC is removed is an implementation consideration that is sometimes left unconfirmed or simply overlooked. In a few instances, recombinant BAC designs have included a Cre-expression cassette that allows for auto-excision of the BAC vector from the herpesvirus genome upon delivery into mammalian cells [8, 11–13]. The current study makes this self-recombining design easier to implement and allows for effective confirmation of auto-excision activity.

As part of this study, we examined the efficiency of BAC excision. Implementing BAC removal using a cell line that stably expresses Cre recombinase in trans required three serial passages to eliminate the BAC from the virus population [7]. This result is intended only as an isolated example of BAC excision efficiency, which likely varies based on the BAC, cell type, and Cre delivery method used in various research settings. Nevertheless, the results underscore that the goal of producing clonal virus populations from BAC clones can be impaired by inadequate BAC vector excision. Although the pYEbac102 clone that served as the progenitor BAC for this study yielded HSV-1 that tolerated the BAC insertion remarkably well when not excised, consistent with prior observations [9], the presence of this extraneous DNA could have undesirable synergistic effects when other genetic modifications are introduced for the study of HSV-1 pathogenesis. Whereas the pHSVF-BFP BAC afforded improved monitoring of BAC excision to help alleviate these concerns, the conversion of this construct to the self-excising design, pHSVF-CREin, eliminated the need to implement a BAC excision protocol and provided several additional advantages [11, 12]. First, BAC removal was efficient. By using the self-excising design approach, virus genomes escaping recombination continue to express Cre, thus pushing the reaction to completion. Second, screening for BAC removal or performing plaque purification became unnecessary. Third, auto-expression of Cre allowed for BAC transfection into any mammalian cell type, which provides the added flexibility to use cells previously designed to stably trans-complement viruses mutated in essential genes.

## Conclusions

Bacterial artificial chromosomes allow for cloning of large foreign sequences in *E. coli*. In some circumstances, removing the prokaryotic vector sequences from the construct for studies in mammalian cells is beneficial. This is particularly true for full-length infectious clones of herpesviruses, where the vector sequences effectively become a large foreign insert in the replicating viral genome. However, removal of BAC vector sequences can be troublesome to monitor. The En Passant recombination constructs described in this report, pEP-TagBFP-in and pEP-CREin-in, address this problem in two ways. First, pEP-TagBFP-in allows for efficient insertion of a blue-fluorescent protein mammalian expression cassette into BAC vectors, which yields a robust report for the presence of BAC vector sequences in transfected and infected cells. The sequence of TagBFP is sufficiently diverged from other fluorescent proteins that it should not lead to unwanted homologous recombination in BACs modified to express green and red fluorescent proteins, and the blue fluorescence emitted from TagBFP is easily discernable. Insertion of the TagBFP expression cassette also makes the conversion of BACs to self-excising BAC clones more reliable, as the fluorescent emissions from TagBFP provide good indication that the site of insertion in a BAC vector is compatible with transgene expression, and furthermore affords a simple screen when inserting the CREin sequence by means of loss of fluorescence. The second construct, pEP-CREin-in, allows for efficient insertion of the CREin sequences that provides autonomous excision of the BAC vector sequences following delivery into mammalian cells. Applying these tools to a HSV-1 infectious clone resulted in production of clonal virus populations that uniformly lacked vector sequences. These viral stocks are particularly advantageous for use in animal models of pathogenesis, but are generally beneficial for all studies. The methods described here can be applied to pre-existing floxed BAC infectious clones of any herpesvirus, or any other BAC construct that would benefit from self-excision properties.

## Additional files

Additional file 1: Table S1. Primers used in this study. Primer sequences used for BAC recombination and data acquisition presented in Fig. 2. (PDF 820 kb) Additional file 2: Raw Data. Raw data used for the production of Figs. 1, 2 and 3. (XLSX 10 kb)

## Abbreviations

BAC: Bacterial artificial chromosome
BFP: Blue fluorescent protein
BGS: Bovine growth supplement
cat: Chloramphenicol acetyl transferase
CPE: Cytopathic effect
CREin: Cre recombinase gene containing a synthetic intron
DMEM: Dulbecco modified Eagle medium
EDTA: Ethylenediaminetetraacetic acid
EP: En passant
FBS: Fetal bovine serum
HCMV IE: Human cytomegalovirus immediate early promoter
HSV-1: Herpes simplex virus type 1
HSVF: Herpes simplex virus type 1 strain F
P#: Passage number
pA: Polyadenylation sequence
