# Supplementary material for: New tools to convert bacterial artificial chromosomes to a self-excising design and their application to a herpes simplex virus type 1 infectious clone
Source: BMC Biotechnol. 2016 Aug 31;16(1):64. doi: 10.1186/s12896-016-0295-4 (PMC5006514; doi:10.1186/s12896-016-0295-4)
Supplement: Additional file 1: Table S1. — Primers used in this study. Primer sequences used for BAC recombination and data acquisition presented in Fig. 2. (PDF 820 kb) [file 12896_2016_295_MOESM1_ESM.pdf]

Supplemental Table 1: Primers used in this study

Final BAC	Primer	BAC Target	Insert Target
pHSVF-BFP (pGS5923)	GS5840: 5'AAGCTGTCAAACATGAGAATTGGTCGACGGCCCGGGCGGC-TAGTTATTAATAGTAATCAATTAGC<br>GS5841: 5'AGCGAGCGAGACGAACGGTCAGAAGCACATCTCGAGGTCG-TAAGATACATTGATGAGTTTG	pYEbac102 (pGS2695)	pEP-BFP-in (pGS4794)
pHSVF-CREin (pGS6000)	GS5952: 5'AGCTGGTTTAGTGAACCGTCAGATCCGCTAGCGGTCGCCACCATG-CCCAAGAAGAAGAGGAAGGTGTC<br>GS5953: 5'CTATTGCTTTATTTGTAACCATTATAAGCTGCAATAAACAAGTTA-CTAATCGCCATCTTCCAGCAGG	pHSVF-BFP (pGS5923)	pEP-CREin-in (pGS1518)
